# Supplementary material for: A rapid method to simultaneously separate bacterial and eukaryotic RNA during infections reveals increased intracellular expression of Staphylococcus aureus and Shigella flexneri virulence factors
Source: Microbiol Spectr. 2026 Apr 6;14(5):e03745-25. doi: 10.1128/spectrum.03745-25 (PMC13141877; doi:10.1128/spectrum.03745-25)
Supplement: Fig. S1 — Scatter plot. [file spectrum.03745-25-s0001.pdf]

Supplementary Figure 1

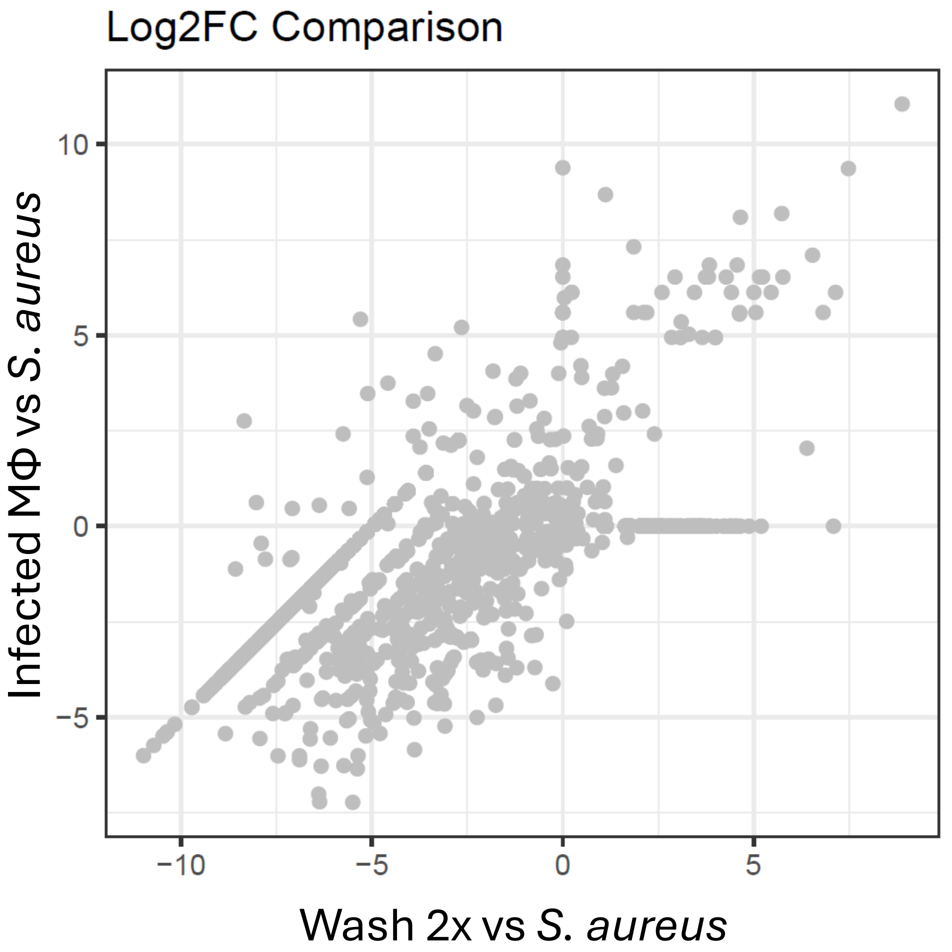

Figure S1. Scatter plot of *S. aureus* RNA from fractionated samples shows good qualitative agreement. Comparison of $\log_2$ fold changes, for two comparisons: Vertical axis: Infected macrophages (infected MΦ) *vs S. aureus* grown in broth culture (*S. aureus*); horizontal axis: and twice washed (Wash 2x) *vs S. aureus* grown in broth culture (*S. aureus*).
